# Systematic review of model-based economic evaluations of heart valve implantations

**DOI:** 10.1007/s10198-017-0880-z

**Published:** 2017-03-06

**Authors:** Simone A. Huygens, Johanna. J. M. Takkenberg, Maureen P. M. H. Rutten-van Mölken

**Affiliations:** 1 Department of Cardiothoracic Surgery, Erasmus University Medical Center, PO Box 2040, 3000 CA Rotterdam, The Netherlands; 2 Department of Health Policy and Management/Institute for Medical Technology Assessment, Erasmus University Rotterdam, Bayle Building, Campus Woudestein, PO Box 1738, 3000 DR Rotterdam, The Netherlands

**Keywords:** Systematic review, Decision-analytic model, Economic evaluation, Heart valve implantations, I190

## Abstract

**Objective:**

To review the evidence on the cost-effectiveness of heart valve implantations generated by decision analytic models and to assess their methodological quality.

**Methods:**

A systematic review was performed including model-based cost-effectiveness analyses of heart valve implantations. Study and model characteristics and cost-effectiveness results were extracted and the methodological quality was assessed using the Philips checklist.

**Results:**

Fourteen decision-analytic models regarding the cost-effectiveness of heart valve implantations were identified. In most studies transcatheter aortic valve implantation (TAVI) was cost-effective compared to standard treatment (ST) in inoperable or high-risk operable patients (ICER range 18,421–120,779 €) and in all studies surgical aortic valve replacement (SAVR) was cost-effective compared to ST in operable patients (ICER range 14,108–40,944 €), but the results were not consistent on the cost-effectiveness of TAVI versus SAVR in high-risk operable patients (ICER range: dominant to dominated by SAVR). Mechanical mitral valve replacement (MVR) had the lowest costs per success compared to mitral valve repair and biological MVR. The methodological quality of the studies was moderate to good.

**Conclusion:**

This review showed that improvements can be made in the description and justification of methods and data sources, sensitivity analysis on extrapolation of results, subgroup analyses, consideration of methodological and structural uncertainty, and consistency (i.e. validity) of the models. There are several opportunities for future decision-analytic models of the cost-effectiveness of heart valve implantations: considering heart valve implantations in other valve positions besides the aortic valve, using a societal perspective, and developing patient-simulation models to investigate the impact of patient characteristics on outcomes.

**Electronic supplementary material:**

The online version of this article (doi:10.1007/s10198-017-0880-z) contains supplementary material, which is available to authorized users.

## Introduction

The first cost-effectiveness analysis on heart valve implantations was published by Wu et al. [1]. They estimated the cost-effectiveness of surgical aortic valve replacement (SAVR: replace native heart valve with a prosthetic heart valve during open heart surgery) compared to standard treatment (ST: often medical management) and found that SAVR was cost-effective [1]. The number of cost-effectiveness analyses on heart valve implantations increased after the introduction of an alternative treatment for severe aortic valve stenosis: transcatheter aortic valve implantation (TAVI: prosthetic heart valve implanted with a catheter, no open heart surgery required).

In 2010, the first model-based cost-effectiveness analysis of TAVI compared to ST and SAVR concluded that TAVI had high potential to be cost-effective for inoperable patients, but the cost-effectiveness of patients with lower operable risk was uncertain [2]. Healthcare decision makers required further evidence on the clinical effectiveness of TAVI to make a reimbursement decision. The Placement of Aortic Transcatheter Valves (PARTNER) trial was the first randomized controlled trial for TAVI [3, 4]. Based on the PARTNER trial results, in 2012 the National Institute for Health and Care Excellence approved reimbursement of TAVI for inoperable patients in the UK but reimbursement for operable patients is still under review [5].

Since then almost every cost-effectiveness analysis investigating TAVI based their clinical effectiveness parameters on the PARTNER trial. There are two trial-based cost-effectiveness analyses [6, 7]; the other cost-effectiveness analyses are based on decision-analytic models. Decision-analytic models represent an explicit way to synthesize evidence on the outcomes and costs of alternative interventions [8].

We are currently developing a decision-analytic model to estimate the cost-effectiveness of current and future heart valve interventions (e.g. tissue-engineered heart valves) [9]. In this light, careful review of existing decision-analytic models addressing related problems is a prerequisite [10].

The goal of this study is to investigate the opportunities for new decision-analytic models in the field of heart valve interventions and to learn from the methodological choices made by previous model developers. Therefore, and in contrast with previous reviews [11–13], we focus on decision-analytic models and exclude cost-effectiveness analyses alongside clinical trials. Furthermore, we are not only interested in decision-analytic models investigating the cost-effectiveness of SAVR and TAVI but we also include decision-analytic models for other heart valve implantations.

## Methods

### Search strategy and selection criteria

This systematic review was conducted according to PRISMA guidelines [14]. On May 28, 2015 several databases were searched (Electronic supplementary material: Appendix 1). Two reviewers (SH & JT or SH & MR) independently determined whether the publications met the inclusion criteria. In case of disagreement, an agreement was negotiated. Publications were included when they reported model-based economic evaluations considering costs and health outcomes of heart valve implantations. Papers solely describing regression models, cost-analyses, non-English publications, conference abstracts, editorials and letters to the editor were excluded. References of selected papers and previous systematic reviews [11–13] were crosschecked for other relevant studies.

### Data extraction

Study and model characteristics and cost-effectiveness results were extracted. Costs were inflated to 2015 and converted to euros(€) using purchaser power parities and exchange rates [15, 16].

### Cost-effectiveness thresholds

Reported cost per quality adjusted life years (QALY) ratios were compared to thresholds used in individual studies and thresholds based on the WHO-CHOICE approach where interventions are highly cost-effective when they have incremental cost-effectiveness ratios (ICER) below the gross domestic product (GDP)/capita, cost-effective if the ICER is 1–3 times the GDP/capita, and not cost-effective when the ICER is more than 3 times the GDP/capita [17, 18].

### Methodological quality assessment

The ‘Drummond checklist’ [19] and ‘Evers checklist’ [20] are often used to appraise methodological quality of economic evaluations conducted alongside clinical trials. Although these checklist are relevant, they are not sufficient to appraise the quality of model based economic evaluations. Therefore, we chose to use the Philips checklist to critically appraise the methodological quality of studies [8]. This checklist is divided into three sections: structure, data and consistency. Within each section criteria can be fulfilled, not fulfilled or not applicable. The checklist was assessed for every study by two reviewers (SH & JT or SH & MR). In case of disagreement, an agreement was negotiated. This assessment had a qualitative nature and studies were not excluded because of low quality scores.

## Results

**Fig. 1:**
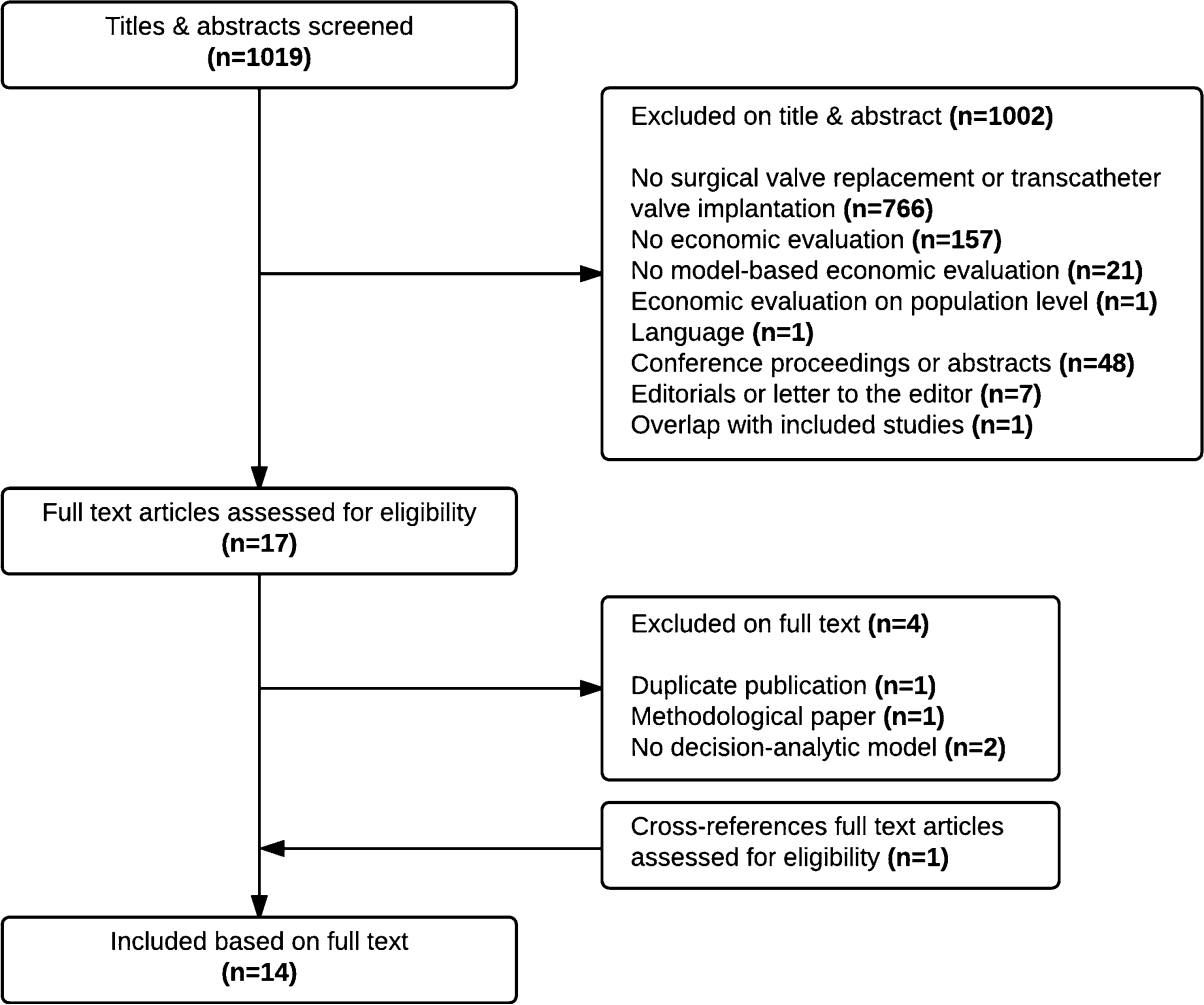
Study selection

The literature search resulted in 1019 studies, of which 14 studies were included (Fig. 1) [2, 21–33].

### Study and model characteristics

Tables 1 and 2 provide an overview of study and model characteristics. Table 1 is structured by valve position and interventions and comparators; TAVI versus ST (often inoperable patients), TAVI versus SAVR (often high operable risk patients), SAVR versus ST (operable patients) and mitral valve repair versus mitral valve replacement (operable patients).

**Table 1:** Study characteristics

Author and year of publication	Target population	Clinical effectiveness data source^c^	Mean patient age		Logistic EuroSCORE		NYHA class III/IV (%)		Intervention of interest	Comparator
			I	C	I	C	I	C		
TAVI versus ST (often inoperable patients)										
SHTG 2010 [2]	Medium risk AS patients: patients for whom there is not currently a clear choice of treatment, as such the choice considered in the analysis is between SAVR, TAVI and MM	REVIVE	70	70	NR	NR	NR	NR	TAVI^b^	MM
	High-risk AS patients: patients who are ineligible for conventional surgery so traditionally get medical management, as such the choice is between TAVI and MM		80	80						
Gada et al. 2012 [25]	High-risk severe AS operable patients: patients with a logistic EuroSCORE >15% and/or STS score >10%	8 registries	82	77	26	21	87	90	TAVI (TF)	MM^a^
Gada et al. 2012 [26]		20 registries	82	81	29	31	77	87	TAVI (TA)	
Neyt et al. 2012 [29]	Inoperable SSAS patients: patients with coexisting conditions associated with a predicted probability of ≥50% of death by 30 days after surgery or a serious irreversible condition. At least two surgeon investigators had to agree that the patient was not a suitable candidate for surgery	PARTNER-B	83	83	26	30	92	94	TAVI (TF)	ST (including MM and/or BAV)
Watt et al. 2012 [33]										
Doble et al. 2013 [23]										
Hancock-Howard et al. 2013 [27]										
Murphy et al. 2013 [28]										
Queiroga et al. 2013 [31]										
Simons et al. 2013 [32]										
Orlando et al. 2013 [30]	Patients unsuitable for SAVR: patients with coexisting conditions associated with a predicted probability of ≥50% of death by 30 days after surgery or a serious irreversible condition. At least two surgeon investigators had to agree that the patient was not a suitable candidate for surgery	PARTNER-B	83	83	26	30	92	94	TAVI^b^	MM
Brecker et al. 2014 [22]	Inoperable and high-risk SSAS patients: Patients considered inoperable or at higher risk for SAVR and anatomically acceptable candidates for elective treatment with the CoreValve System	ADVANCE (all TAVI patients) PARTNER-B (ST patients)	81	83	19	30	80	94	TAVI (TF, direct aortic, or subclavian)	ST (including MM and/or BAV)
		ADVANCE (TAVI patients with >20% logistic EuroSCORE) PARTNER-B (ST patients)	83	83	32	30	85	94		
TAVI versus SAVR (often high-risk operable patients)										
SHTG 2010 [2]	Low-risk AS patients: patients who are assumed to be eligible for SAVR but for whom TAVI could be an alternative	REVIVE	60	60	NR	NR	NR	NR	TAVI^b^	SAVR
	Medium risk AS patients: patients for whom there is not currently a clear choice of treatment, as such the choice considered in the analysis is between SAVR, TAVI and MM		70	70						
Gada et al. 2012 [25]	High-risk severe AS operable patients: patients with a logistic EuroSCORE >15% and/or STS score >10%	8 registries	82	77	26	21	86	90	TAVI (TF)	SAVR
Gada et al. 2012 [26]		20 registries	82	81	29	31	77	87	TAVI (TA)	
Neyt et al. 2012 [29]	High-risk operable SSAS patients: patients with a predicted risk of operative mortality rate of ≥15% or a Society of Thoracic Surgery risk score of ≥10%	PARTNER-A	84	85	29	29	94	94	TAVI (TF + TA)	SAVR
Doble et al. 2013 [23]										
Fairbairn et al. 2013 [24]										
Orlando et al. 2013 [30]	Patients suitable for SAVR: TAVI and MM patients Inoperable SSAS patients from the PARTNER-B trial: patients with coexisting conditions associated with a predicted probability of ≥50% of death by 30 days after surgery or a serious irreversible condition. At least two surgeon investigators had to agree that the patient was not a suitable candidate for surgery SAVR patients Patients undergoing isolated SAVR	PARTNER-B (for TAVI and MM) and two cohort studies [50, 51] (for SAVR)	83	NR	29	10–20	92	NR	TAVI (TF + TA)	SAVR (90%) MM (10%)
SAVR versus ST (operable patients)										
SHTG 2010 [2]	Medium risk AS patients: patients for whom there is not currently a clear choice of treatment, as such the choice considered in the analysis is between SAVR, TAVI and MM	REVIVE	70	70	NR	NR	NR	NR	SAVR	MM
Gada et al. 2012 [25]	High-risk severe AS operable patients: patients with a logistic EuroSCORE >15% and/or STS score >10%	8 registries	82	77	26	21	86	90	SAVR	MM^a^
Gada et al. 2012 [26]		20 registries	82	81	29	31	77	87		
Mitral valve repair versus replacement (operable patients)										
Beresniak et al. 2013 [21]	Patients with mitral valve disease undergoing surgical mitral valve repair or replacement	Cohort study of the Georges Pompidou European Hospital	NR	NR	NR	NR	NR	NR	Surgical mitral valve repair	Surgical mitral valve replacement

*I* intervention of interest, *C* comparator, *NR* not reported, *SSAS* severe symptomatic aortic stenosis, defined as an aortic valve area 0.8 cm^2^ with either a mean valve gradient > 40 mm Hg or a peak jet velocity > 4.0 m/s. *AS* aortic stenosis, *SAVR* surgical aortic valve replacement, *TAVI* transcatheter aortic valve replacement, *TF* transfemoral, *TA* transapical, *MM* medical management, *ST* standard therapy, including MM and/or balloon aortic valvuloplasty (BAV). *NYHA class* New York Heart Association class. *PARTNER-A* comparing TAVI with SAVR in high-risk operable patients [3]. *PARTNER-B* comparing TAVI with MM/ST in inoperable patients [4]. *REVIVE* the Registry of Endovascular Implantation of Valves in Europe trial started in 2003 in a single centre in France with the aim of studying the feasibility and safety of TAVI in inoperable patients [52]. *ADVANCE* multicentre, non-randomized study that included 44 centres in 12 countries evaluating the outcomes of a self-expanding transcatheter aortic valve system in patients considered inoperable or at a higher surgical risk [53]

^a^Medical management comprised antithrombotic therapy for treatment of concomitant coronary artery disease or atrial fibrillation, antihypertensive drugs in case of arterial hypertension, statins for treatment of hypercholesterolemia, and diuretics for management of heart failure symptoms, rarely complemented by digoxin [54]

^b^Implantation route not defined

^**c**^The sources of other data types (mortality, resource use, costs and utilities) can be found in Table A2.2 in the Electronic supplementary material

**Table 2:** Model characteristics

Author and year of publication	Model type	Health states	Time horizon	Cycle length	Discount rate	Study perspective	Country
SHTG 2010 [2]	Decision tree; Markov model	Short-term: dead, alive, major (assumed to result in failure of the valve implantation with the patient left in a state no better than their original manifestation of AS), minor (assumed to resolve with appropriate medical care), or no procedure related event, convert to SAVR, convert to MM, AS/failed valve replacement, and functioning valve replacement Long-term: AS/failed valve replacement, procedure related event, functioning valve replacement, death	1 month; until the majority of patients have died	N/A; 1 year	C: 3.5% E: 3.5%	Healthcare	UK
Gada et al. 2012 [25]	Markov model	Medical management, screened for TAVI, SAVR and peri-procedural risks, TAVI and peri-procedural risks, post-SAVR or TAVI complication (including endocarditis, hemorrhage, valve thrombosis, and non-cerebral), heart failure, stroke, dead	Lifetime	1 year	C: 5% E: –	Healthcare payer	US
Gada et al. 2012 [26]							
Neyt et al. 2012 [29]	Markov model	Mortality, hospitalization, other events (repeat hospitalization, minor/major stroke and TIA, and cardiac re-interventions), and no event	Lifetime/ 1 year^a^	1 month	C: 3% E: 1.5%	Healthcare	Belgium
Watt et al. 2012 [33]	Two interlinked Markov models	Short-term: ICU non-ICU, home care, post-hospital rehabilitation (community and managed) and death Long-term: home care, reoperation and death	1 month; 10 years	1 day; 1 month	C: 3.5% E: 3.5%	Healthcare	UK
Beresniak et al. 2013 [21]	Decision tree	Sequential treatment switches allowed at each 5-year interval in case of failure of the former treatment option	10/20 years	N/A	C: – E: –	Healthcare	France
Doble et al. 2013 [23]	Decision tree; Markov model	Short-term: alive without complications, other acute complications (endocarditis, major vascular complications, paravalvular leaks, PI, major bleeding, AF), stroke (temporary or permanent disability), MI, AKI (no, temporary, and permanent dialysis), reoperation, conversion to SAVR, cumulative death Long-term: alive without complications, stroke first year, stroke subsequent years, MI first year, MI subsequent years, post-AKI, alive and death after complications, and death	1 month; 20 years	N/A; 1 year	C: 5% E: –	Healthcare	Canada
Fairbairn et al. 2013 [24]	Decision tree; Markov model	Short-term: after TAVI/SAVR transition to NYHA class I-IV or dead Long-term: transitions from NYHA class I-IV to dead	2 years; 10 years	N/A; 1 year	C: 3.5% E: 3.5%	Healthcare	UK
Hancock-Howard et al. 2013 [27]	Decision tree	After treatment: alive or dead. When alive: early or no early complication. After both these health states: late complication (major stroke with full recovery, major stroke with ongoing care and no stroke) or no late complication. Complications in no stroke: valve thromboembolism, PI, endocarditis, reoperation, MI, renal failure, BAV, hospital readmission, SAVR. In addition to these complications, other complications were only considered early: major access site/vascular complication, major paravalvular leak, and arrhythmia/atrium fibrillation	3 years	N/A	C: 5% E: 5%	Healthcare	Canada
Murphy et al. 2013 [28]^b^	Decision tree; Markov model	Short-term: dead, alive, major (e.g. valve thromboembolism or MI: long-term effect), minor (e.g. PI or vascular events: short-term effect), or no procedure related event, convert to SAVR, convert to MM, AS/failed valve replacement, and functioning valve replacement Long-term: AS/failed valve replacement, procedure related event, functioning valve replacement, and death	1 month; Lifetime	N/A; 1 year	C: – E: –	Healthcare	UK
Orlando et al. 2013 [30]	Decision tree	Suitable for surgery followed by SAVR, TAVI (when available) and MM. Not suitable for surgery followed by TAVI (when available) and MM. After treatment: hospital-free survival and other survival (surviving patients who had undergone ≥1 episode of hospitalization after initial treatment)	1 month; 25 years	N/A	C: 3.5% E: 3.5%	Healthcare	UK
Queiroga et al. 2013 [31]	Markov model	Survival and death	5 years	3 months	C: 5% E: 5%	Healthcare	Brazil
Simons et al. 2013 [32]	Markov model	Health states based on combination symptom status (NYHA class I/II or III/IV) and major complications (stroke, vascular complication, bleed)	Lifetime	1 month	C: 3% E: 3%	Healthcare^c^	US
Brecker et al. 2014 [22]^d^	Two interlinked Markov models	Short-term: ICU, non-ICU, home care, post-hospital rehabilitation (community and managed) and death Long-term: home care, reoperation and death	1 month; 5 years	1 day; 1 month	C: 3.5% E: 3.5%	Healthcare	UK

*C* costs, *E* effects, *N/A* not applicable, *AS* aortic stenosis, *SAVR* surgical aortic valve replacement, *TAVI* transcatheter valve implantation, *BAV* balloon aortic valvuloplasty, *MM* medical management, *ICU* intensive care unit, *PI* pacemaker implantation. *AF* atrial fibrillation, *MI* myocardial infarction, *AKI* acute kidney injury, *TIA* transient ischemic attack, *NYHA* New York Heart Association, *Healthcare perspective* includes all direct healthcare costs regardless of who pays them, *Healthcare payer perspective* includes all direct healthcare costs covered by the health insurer or the NHS (i.e. the amount of costs reimbursed to the provider)

^a^The time horizon is lifetime in the model comparing TAVI with ST in inoperable patients and 1 year in the model comparing TAVI versus SAVR in high-risk operable patients

^b^Based on model of SHTG [2]

^c^Societal perspective according to authors, but costs outside of healthcare are not taken into account

^d^Same model as Watt et al. [33]

### Cost-effectiveness outcomes

Table 3 shows the cost-effectiveness outcomes structured by valve position and interventions and comparators.

**Table 3:** Cost-effectiveness outcomes

Author and year of publication	Subgroups	Health outcomes				Costs in 2015 € (PPPs)			Cost-effectiveness		WTP threshold	
			TAVI (absolute)	ST (absolute)	TAVI vs ST (incremental)	TAVI (absolute)	ST (absolute)	TAVI vs ST (incremental)	ICER as reported	ICER in 2015 € (PPPs)	Individual studies	WHO approach in 2015 € (PPPs)^a^
TAVI versus ST (often inoperable patients)												
SHTG 2010 [2]	Medium-risk	QALY	2.9	1.53	1.37	46,690	20,253	26,436	NR	NR	£30,000	125,199
	High-risk	QALY	2.18	1.53	0.65	41,548	20,258	21,290	£22,603	32,774		
Gada et al. 2012 [25]		QALY	1.78	NR	NR	58,193	NR	NR	US$ 39,964	39,084	US$ 100,000	168,198
Gada et al. 2012 [26]		QALY	1.66	NR	NR	54,477	NR	NR	US$ 44,384	42,622	US$ 100,000	168,198
Neyt et al. 2012 [29]		QALY	NR	NR	0.74	NR	NR	38,751	44,900 €	52,407	Based on UK: 22,800–34,200 €	137,727
		LY	NR	NR	0.88	NR	NR	38,751	42,600 €	49,722		
Watt et al. 2012 [33]		QALY	2.36	0.80	1.56	43,125	7140	35,985	£16,200	23,133	£20,000	125,199
Doble et al. 2013 [23]		QALY	NR	NR	0.60	70,227	45,742	24,486	CDN$ 51,324	40,502	CDN$ 50,000	132,891
		LY	NR	NR	0.85	70,227	45,742	24,486	CDN$ 36,458	28,771		
Hancock-Howard et al. 2013 [27]		QALY	1.33	0.84	0.49	47,376	34,641	12,735	CDN$ 32,170	26,117	CDN$ 20,000–100,000	132,891
Murphy et al. 2013 [28]		QALY	1.63	1.19	0.44	38,685	16,786	21,899	£35,956	49,569	£20,000–30,000	125,199
		LY	2.54	2.24	0.30	38,685	16,786	21,899	NR	NR		
Orlando et al. 2013 [30]		QALY	2.85	0.98	1.87	39,745	5265	34,480	£12,900	18,421	£20,000–30,000	125,199
Queiroga et al. 2013 [31]		LY	2.5	1.53	0.97	71,245	20,742	50,503	R$ 90,161	52,215	based on US: R$ 100,000	NA
Simons et al. 2013 [32]	Without BAV	QALY	1.93	1.19	0.73	168,791	83,447	85,444	US$ 116,500	116,287	$100,000	168,198
		LY	2.93	2.08	0.86	168,791	83,447	85,444	US$ 99,900	99,718		
	With ≥ 1 BAV	QALY	1.93	1.24	0.69	168,791	86,142	82,649	US$ 121,000	120,779		
		LY	2.93	1.97	0.96	168,791	86,142	82,649	US$ 85,700	85,544		
Brecker et al. 2014 [22]	All patients	QALY	2.29	0.78	1.51	46,256	17,795	28,461	£13,943	18,863	£20,000	125,199
	Patients with > 20% logistic EuroSCORE	QALY	2.02	0.78	1.24	47,524	17,749	29,775	£17,718	23,970		

Author and year of publication	Subgroups		TAVI (absolute)	SAVR (absolute)	TAVI vs. SAVR (incremental)	TAVI (absolute)	SAVR (absolute)	TAVI vs. SAVR (incremental)	ICER as reported	ICER in 2015 € (PPPs)	Individual studies	WHO approach in 2015 € (PPPs)^a^
TAVI versus SAVR (often high-risk operable patients)												
SHTG 2010 [2]	Low-risk	QALY	3.71	3.65	0.06	51,942	45,004	6939	£87,293	124,652	£30,000	125,199
	Medium-risk	QALY	2.90	2.82	0.08	45,981	38,167	7814	£72,412	103,402		
Gada et al. 2012 [25]		QALY	1.78	1.72	0.06	58,193	55,099	3094	US$ 52,773	51,611	US$ 100,000	168,198
Gada et al. 2012 [26]		QALY	1.66	1.70	−0.04	54,477	54,381	96	Dominated	Dominated	US$ 100,000	168,198
Neyt et al. 2012 [29]		QALY	NR	NR	0.03	NR	NR	23,807	Around 750,000 €	Above €750,000	Based on UK: 22,800–34,200 €	137,727
Doble et al. 2013 [23]		QALY	NR	NR	−0.10	67,674	58,872	8801	Dominated	Dominated	CDN$ 50,000	132,891
		LY	NR	NR	0.01	67,674	58,872	8801				
Fairbairn et al. 2013 [24]		QALY	2.81	2.75	0.06	72,505	74,366	−1862	Dominant	Dominant	£20,000	125,199
Orlando et al. 2013 [30]		QALY	2.85	3.46	−0.61	39,745	28,375	11,370	Dominated	Dominated	£20,000–30,000	125,199

Author and year of publication	Subgroups		SAVR (absolute)	ST (absolute)	SAVR vs. ST (incremental)	SAVR (absolute)	ST (absolute)	SAVR vs. ST (incremental)	ICER as reported	ICER in 2015 € (PPPs)	Individual studies	WHO approach in 2015 € (PPPs)^a^
SAVR versus ST (operable patients)												
SHTG 2010 [2]	Medium-risk	QALY	2.82	1.53	1.29	38,167	19,946	18,221	£9880	14,108	£30,000	125,199
Gada et al. 2012 [25]		QALY	1.72	NR	NR	55,099	NR	NR	US$ 39,280	38,415	US$ 100,000	168,198
Gada et al. 2012 [26]		QALY	1.70	NR	NR	54,381	NR	NR	US$ 42,637	40,944	US$ 100,000	168,198

Author and year of publication			Repair	Replacement		Repair	Replacement		Costs/success repair	Costs/success replacement		
			(Absolute)	Biological (absolute)	Mechanical (absolute)	(Absolute)	Biological (absolute)	Mechanical (absolute)		Biological	Mechanical	
Mitral valve repair versus mitral valve replacement (operable patients)												
Beresniak et al. 2013 [21]	10 years time horizon	Succes rate	88.3	71.7	70.4	31,414	35,501	38,499	41,773	58,138	64,212	
	20 years time horizon	Succes rate	33.4	30.2	51.6	33,457	44,632	48,956	117,619	173,531	111,402	

*NR* not reported, *NA* not available, *SAVR* surgical aortic valve replacement, *TAVI* transcatheter valve implantation, *BAV* balloon aortic valvuloplasty, *MM* medical management, *ICER* incremental cost-effectiveness ratio, *QALY* quality-adjusted life years, *LY* life years, *WTP* willingness-to-pay, *PPP* purchasing power parities

^**a**^Three times GDP/capita of country of interest

#### TAVI versus ST (often inoperable patients)

The costs of TAVI compared to ST were higher, but QALYs gained were also higher. According to thresholds used in individual studies, TAVI is cost-effective compared to ST in eight studies [2, 22, 25–27, 30, 31, 33] and not cost-effective in four studies [23, 28, 29, 32]. When applying the WHO-CHOICE approach, TAVI is cost-effective compared to ST in all studies and even highly cost-effective (ICER < GDP/capita) in seven studies [2, 22, 25–27, 30, 33].

#### TAVI versus SAVR (often high-risk operable patients)

TAVI was dominated by SAVR (i.e. higher costs, lower QALY gain) in three studies [23, 26, 30], high ICERs were reported in three studies [2, 25, 29], and TAVI was dominant in one study [24] (i.e. lower costs, higher QALY gain). According to thresholds used in individual studies, TAVI was not cost-effective in two of three studies where TAVI was not dominant or dominated by SAVR [2, 29]. Using the WHO-CHOICE approach, TAVI was not cost-effective compared to SAVR in Neyt et al. [29], and TAVI was cost-effective in the SHTG report [2] and in Gada et al. [25].

#### SAVR versus ST (operable patients)

SAVR gains more QALYs at higher costs than ST. According to thresholds used in individual studies and the WHO-CHOICE approach SAVR is (highly) cost-effective compared to ST in all studies.

#### Mitral valve repair versus mitral valve replacement (operable patients)

One study evaluated heart valve implantations in the mitral valve position [21]. They found that mechanical mitral valve replacement has the lowest costs per success (when using a 20-year time horizon). To compare these results with heart valve implantations in other valve positions and to assess whether it falls below the cost-effectiveness threshold, the effects should be expressed in QALYs.

### Methodological quality assessment

The assessment of methodological quality of studies using the Philips checklist is reported in Table A2.1 in the Electronic supplementary material. The total score represents the percentage of criteria that were fulfilled, corrected for criteria that were not applicable, and ranged from 49 to 87%. The lowest percentage was found in the study on mitral valve interventions [21].

## Discussion

### Cost-effectiveness outcomes

Even though most studies compared the same heart valve implantations, cost-effectiveness results varied substantially between studies. Based on thresholds from individual studies or using the WHO-CHOICE approach, TAVI was cost-effective compared to ST in inoperable or high-risk operable patients in most studies and in all studies SAVR was cost-effective compared to ST in operable patients. The results were not consistent on the cost-effectiveness of TAVI versus SAVR in high-risk operable patients, ranging from TAVI being dominant to being dominated by SAVR. However, the cost-effectiveness thresholds were relatively high. The thresholds used in individual studies ranged from £20,000/QALY to CDN$100,000/QALY and thresholds based on the WHO-CHOICE approach ranged from 123,264 €/QALY for France to 168,198 €/QALY for the US. When we apply the threshold of the UK (£30,000 ≈ €43,000/QALY), TAVI is cost-effective compared to ST in seven instead of eight (according to thresholds used in individual studies) or all (according to WHO-CHOICE approach) studies. Just as with the individual studies' and WHO approach thresholds, SAVR is cost-effective compared to ST in all three studies. Using the UK threshold does not influence our conclusion on the cost-effectiveness of TAVI versus SAVR; it remains not cost-effective in all but one study.

Our results did not reflect a clear trend in the cost-effectiveness of heart valve implantations over time; probably due to the short time frame in which the studies were performed (>80% in 2012–2013).

### Methodological quality assessment

There was no correlation between methodological quality scores and ICERs of the included studies (Spearman’s rank correlation coefficients: TAVI vs ST (12 studies) = 0.000, TAVI vs SAVR (7 studies) = −0.126, SAVR vs ST: correlation not determined because there were only three studies in this subgroup). The methodological quality assessment showed that the decision-analytic models were of moderate to good quality. However, authors did not always justify their choices and assumptions and major improvements can be made in the description of methodology. The following discusses our assessment of the methodological quality, structured according to the Philips checklist [8].

#### Perspective

Most studies used a healthcare perspective (i.e. include all direct healthcare costs) and two studies used a healthcare payer perspective (i.e. only includes healthcare costs covered by the health insurer or the NHS) [25, 26]. Simons et al. [32] claimed to use a societal perspective while only healthcare costs were included. Contrary to our expectations, studies performed from a healthcare payer perspective did not report significantly lower costs. However, it is possible that the studies performed from a healthcare payer perspective underestimated the costs of TAVI because they both assume that payers would provide the same reimbursement for the TAVI and SAVR procedure and subsequent hospitalisation [25, 26].

The ICERs are generally the lowest in the UK and the highest in the US. Comparisons of studies within the US showed that the costs of TAVI in Gada et al. [25, 26] are considerably lower than in Simons et al. [32], probably due to the healthcare payer perspective of Gada et al. compared to the healthcare perspective of Simons et al., the assumption of same procedure costs for TAVI and SAVR in Gada et al. while TAVI is, in general, more expensive, and/or difference in operable risks (high-risk operable patients in Gada et al. vs inoperable patients in Simons et al.).

#### Rationale for structure

Many studies combined a short- (often 1 month) and long-term model, mostly decision trees and Markov models. Health states were based on treatment [21], ward or site where care was provided [22, 33], New York Heart Association (NYHA) class [24], complications [2, 23, 25–29], survival [31], or a combination of NYHA class and treatment or complications [30, 32]. In our view, two studies chose a too simplistic model structure only including health states of survival and death [31] or NYHA classes and death [24] without explicitly including valve-related complications. The simple model structure did not result in divergent results compared to other studies in Queiroga et al. [31], but Fairbairn et al. [24] found that TAVI is dominant while all other studies comparing TAVI with SAVR found high ICERs or that TAVI was dominated by SAVR.

Only one study described who was involved in developing the model structure [33]. Two studies reported information about developing the model structure [22, 32], but they did not explicitly discuss this process nor referred to an underlying conceptual model. Cooper et al. also found that few studies (10%) report the development process of the model structure [34]. Transparency of model development is important to assess to what extent model development is based on clinical considerations and/or considerations regarding data availability of model parameters [10].

#### Structural assumptions

Several structural assumptions were not reasonable and some might have impacted the cost-effectiveness results. For instance, four studies assumed that valve prosthesis functionality and/or complication rates were similar for TAVI and SAVR [25, 26, 33] or assumed TAVI valves retain functionality during the patient’s lifetime [24]. These assumptions might over- or underestimate the effects of TAVI, because several studies found significant differences in post-procedure complications between TAVI and SAVR [3, 35]; and since TAVI is a relatively new procedure the long-term effects are unclear.

Further, Orlando et al. [30] assumed that TAVI and ST patients in the state ‘survival with ≥ 1 episode of hospitalisation after initial treatment’ have the same costs and QALY outcomes, regardless of how many further hospital admissions occur. If the frequency of further admissions and reasons for admissions (and thus costs and quality of life) are different between TAVI and ST patients, this assumption leads to bias in cost-effectiveness outcomes which might explain the relatively low ICER reported in this study [30].

#### Strategies and comparators

Many studies evaluated TAVI, but the implantation routes differed. Most studies investigated transfemoral TAVI (through the leg), while others investigated transapical TAVI (through the chest cavity), or combinations of implantation routes. Further, almost all studies investigated balloon-expandable transcatheter valve prostheses, while one study [22] evaluated self-expanding transcatheter valve prostheses. There was no clear trend in cost-effectiveness outcomes of studies considering different implantation routes or types of prostheses. However, two studies using comparable methods to determine the cost-effectiveness of both implantation routes reported a more favourable ICER for transfemoral than transapical TAVI compared to ST and SAVR [25, 26]. This might be explained by higher disease severity of patients undergoing transapical TAVI; which are often patients with a porcelain aorta who are not eligible for transfemoral TAVI.

The definition of ‘standard treatment (ST)’ or ‘medical management (MM)’ differed between studies. In studies based on the PARTNER trial [22, 23, 27–29, 31–33] ST includes MM and is combined with balloon aortic valvuloplasty (BAV) in more than 80% of patients. In other studies the comparator is MM without BAV. The ICERs of studies considering sole MM are not clearly different from studies considering ST as comparator. However, Simons et al. [32] performed separate analyses for TAVI compared to ST with and without BAV and found a more favourable ICER for TAVI compared to ST without BAV than with BAV [32].

#### Time horizon

The appropriate time horizon when evaluating the cost-effectiveness of heart valve implantations is lifetime, because the interventions affect mortality rates [36]. Although the time horizons of the studies might seem different, time horizons of 10 years or longer are equivalent to lifetime because of the high age of patients undergoing valve replacement (±80 years). In four studies the time horizon is too short (1–5 years) to capture all relevant differences between interventions [22, 27, 29, 31]. There was no clear association between time horizon and cost-effectiveness outcomes, except for the study of Neyt et al. who reported a high ICER of TAVI compared to SAVR, that might be explained by the short time horizon (1 year) during which the high procedure costs cannot be compensated with potential increased life expectancy [29].

#### Cycle length

Common practice after heart valve implantations is to schedule follow-up visits at least once a year [37]. Therefore, the appropriate cycle length should be 1 year or shorter. This was the case in all studies, except for one study that used a cycle length of 5 years [21].

#### Data identification

Several studies failed to describe their data sources in such detail that replication of the study using the same data would be possible [21, 25, 26]. Especially methods of deriving expert opinion and choices of data sources were unclear.

#### Data modelling: baseline data

Since TAVI is a relatively new treatment, (real-world) clinical effectiveness data are limited. Therefore, many studies used the PARTNER trial as source for clinical data. This trial consists of two cohorts: PARTNER-A comparing TAVI with SAVR in high-risk operable patients [3] and PARTNER-B comparing TAVI with ST in inoperable patients [4]. Even though many studies used clinical data from these cohorts, there are considerable differences in resulting cost-effectiveness outcomes. Possible explanations for these differences are inclusion of other cost components or sources, other methods of extrapolation of survival or utilities beyond the follow-up time of the trial, variations in time horizon, different model structures, included complications, etc. [25, 26, 38, 39]. The baseline characteristics of populations differed between studies, especially operable risk. Most studies comparing TAVI with ST included inoperable patients based on the PARTNER-B trial definition [23, 27–33], while patients in other studies were at lower operable risk [2, 22, 25, 26]. The latter studies had lower mean patient ages and fewer patients in NYHA class III/IV, but they did not report better cost-effectiveness outcomes [2, 22, 25, 26].

Three studies comparing TAVI with SAVR included high-risk operable patients based on the PARTNER-A trial definition [23, 24, 29]. Other studies used slightly different definitions, resulting in the inclusion of patients with lower mean age, logistic EuroSCORE and/or proportion of patients in NYHA class III/IV [2, 25, 26, 30]. Most of these studies found that TAVI costs more, but gains more QALYs, while studies using the PARTNER-A trial definition found that TAVI is dominated by SAVR.

Besides differences between studies, there were differences in baseline characteristics between groups within studies that might have influenced the cost-effectiveness outcomes [22, 30]. For example, Orlando et al. [30] derived survival estimates from different sources with lower operable risks for SAVR patients compared to TAVI. Therefore, SAVR patients survival may be overestimated, resulting in lower incremental QALY gains due to TAVI. Further, Neyt et al. [29] based costs of SAVR on patients with a lower surgical risk (i.e. >70 years with high severity of illness index, but not selected on operable risk) than the TAVI patients. This might explain the high incremental costs of TAVI in this study. In addition, there are unmeasured patient characteristics that are not considered in operable risk scores, such as patient frailty, that are important in treatment selection [40]. Consequently, this might have resulted in other unobservable differences in patient characteristics between SAVR and TAVI patients that may have influenced the results.

#### Data modelling: treatment effects

The time horizon of most models included in this review is (equivalent to) lifetime, while the follow-up of the clinical trials that are used as input for mortality and complication rates is limited to a few years. Therefore, the included studies needed to make assumptions about survival beyond the trial data, or needed to extrapolate the available data using survival analysis techniques. The extrapolation technique of survival data was reported in most studies (except for Beresniak et al. [21] and Gada et al. [25, 26]), but there was a lack of consistency in techniques between studies which might have influenced cost-effectiveness outcomes.

Three studies explicitly stated using separate parametric models to fit survival curves for TAVI versus ST because the proportional hazard assumption did not hold [22, 30, 33]. Brecker et al. [22] and Orlando et al. [30] used a Weibull distribution, but it was not reported which parametric function Watt et al. [33] used. The all-cause mortality increases faster over time in ST than TAVI patients [22], which might explain the relatively high incremental QALY gains of TAVI in these studies [22, 30, 33].

Queiroga et al. [31] also fitted a Weibull distribution to the observed values, but it is unclear whether separate functions were fitted for both treatment groups. Further, Simons et al. [32] used a piecewise exponential curve accounting for higher mortality rates in ST during the first 6 months than the period thereafter, while other studies continued the trend of higher mortality beyond 6 months. This would result in a higher QALY gain after ST in Simons et al. compared to other studies, which was true for five of the other seven studies that reported LY (life years) or QALY gain after ST [22, 27, 28, 30, 33].

Other studies seem to have assumed that the proportional hazard assumption was true from the time of the intervention until death. Fairbairn et al. [24] assumed the same constant proportional changes observed from year 1 to year 2 for the years beyond two years after the intervention. Hancock-Howard et al. [27] extrapolated the 1-, 6-, 12- and 24-month survival data from the PARTNER trial to 36 months using an exponential trend line function. Neyt et al. [29] assumed that the difference between life expectancy of TAVI and MM patients remained constant during the lifetime horizon of the model and after 1 year the monthly mortality rate increased according to age- and sex- adjusted mortality rates of the general population. As expected, these studies reported smaller incremental QALY differences compared to studies using separate parametric models for different treatments [22, 30, 33].

Doble et al. [23] based the mortality rates from 2 to 20 years after the intervention on Canadian life tables. This means that they assume that the intervention has no continuing effect beyond 2 years after the intervention. This might explain the small difference in life years after SAVR and TAVI found in this study (0.01 LY).

Two studies modelled the mortality rate by multiplying the age- and sex-adjusted mortality rates of the general population with 1.5 to represent higher than average mortality risk in TAVI patients, whereas the life expectancy of MM patients was assumed to be 3 years [2, 28]. This means that the mortality rate in TAVI patients was 50% higher than the average population, which might explain the low incremental QALY gain reported in (the high-risk subgroup of) these studies.

#### Data modelling: costs

Most studies discounted costs and effects according to national economic evaluation guidelines, but there were four studies that did not report whether and how costs and effects were discounted [21, 25, 26, 28]. Discount rates did not seem to influence cost-effectiveness outcomes much, suggesting other differences between studies had a larger impact on results.

There has been much debate on including costs unrelated to the disease or intervention of interest during life years gained [41]. Simons et al. [32] were the only study that included additional healthcare costs unrelated to aortic stenosis or its treatment and management. Since the hazard rate of death is higher in patients in NYHA class I/II that received MM with BAV compared to TAVI [32], these additional healthcare costs are mostly accrued by TAVI patients. This might explain the relative high ICER found in this study. This finding is in line with another study that illustrated that including unrelated medical costs would increase the ICER of TAVI versus ST [41].

#### Data modelling: quality of life weights (utilities)

The way to translate PARTNER trial data to utilities differed between studies resulting in different utility estimates. Seven studies [2, 22–24, 28, 30, 33] calculated utilities based on utilities per NYHA class derived from other literature [42–45] multiplied with the proportion of patients in each NYHA class in the PARTNER trial. The NYHA class consists of four classes reflecting the patient’s limitations during physical activity. In contrast with general quality of life instruments, the NYHA class is assessed by clinicians instead of patients and does not consider social and mental/emotional aspects of quality of life [46]. In addition, applying utilities by NYHA class might underestimate the uncertainty in utility estimates because a change in NYHA class is associated with a fixed change in utility similar for each patient. This might explain the relatively high incremental QALY gains due to TAVI in two studies [22, 33] that used relatively high fixed utility gains for each lower NYHA class, because 1 year after the intervention a larger proportion of TAVI patients compared to ST patients was in a lower NYHA class [4]. Furthermore, utility estimates varied substantially between sources; not only in absolute value for the same NYHA class, but also in the differences between NYHA classes [47]. Therefore, indirect utility assessment using NYHA class is inappropriate and direct utility assessment using preference-based quality of life instruments is preferred. However, we found no clear difference in utility estimates based on NYHA classes or EQ-5D measurements.

There were several other assumptions about utilities that might have influenced cost-effectiveness outcomes of the studies. For example, Orlando et al. [30] made a distinction between utilities of TAVI survivors with and without rehospitalisation, that was not applied to MM patients. Therefore, TAVI patients without rehospitalisation could gain more QALYs than MM patients without rehospitalisation. This might explain the relatively high incremental QALY gain due to TAVI found in this study.

#### Assessment of uncertainty

The quality of a decision-analytic model does not only depend on the methods of determining the point estimate of the ICER, but also on how uncertainty surrounding this outcome is considered [48]. Parameter and structure uncertainty were most often addressed, but most studies could be improved by also considering methodological uncertainty and heterogeneity. Only six studies reported information on statistical significance (*p* values or confidence intervals) of differences in costs and utilities [21, 22, 25, 29, 32, 33]. In all but one study [25] the differences were statistically significant. Twelve studies reported the probability of being cost-effective [2, 22–30, 32, 33] and nine studies supported these probabilities by publishing cost-effectiveness acceptability curves [2, 23, 24, 27–30, 32, 33].

#### Consistency (i.e. validity)

The studies did not pay much attention to consistency of their models. Only three studies [2, 23, 32] reported testing the mathematical logic of their model (internal consistency, e.g. model replication with other software) and two studies calibrated their model against independent data (external consistency) [29, 32]. Further, about half of the studies did not compare their results with previous decision-analytic models [2, 21, 25, 26, 28, 31, 33]. However, when studies were published before 2012 we assumed that it was not possible to compare with previous studies because they did not exist or were published during the time of the study [2, 21, 25, 26].

### Opportunities for future economic models

This review revealed several opportunities for future economic models regarding heart valve implantations.

Firstly, gaps in the literature on model based economic evaluations of heart valve implantations can be filled by evaluating cost-effectiveness of heart valve implantations in valve positions other than the aortic valve and by comparing the cost-effectiveness of SAVR with mechanical or biological valves. Both valve types have their own strengths and limitations and there are differences in healthcare use which might influence cost-effectiveness. Further, it would be interesting to investigate how including costs outside of healthcare (societal perspective), such as productivity and informal care costs, would influence the cost-effectiveness of heart valve implantations.

Secondly, there are methodological alternatives to the frequently used decision trees and Markov models, such as patient-simulation models. Advantages of patient-simulation models are their ability to incorporate recurrent events and to ‘remember patient history’ without producing unmanageable numbers of health states, resulting in greater flexibility in examining the impact of patient characteristics on outcomes [36, 49].

Thirdly, improvements can be made in the methodological quality of studies by describing and justifying chosen methods and data sources in more detail, performing sensitivity analysis on extrapolation of results, performing subgroup analyses, and considering methodological and structural uncertainty and consistency (i.e. validity) of the model.

Finally, in this review only two studies used real-world data from patient registries instead of clinical trials [21, 22]. In the future, we expect more model-based cost-effectiveness studies using data from patient registries including TAVI patients. However, the comparison of TAVI and ST in these registries will become increasingly difficult because of the positive results of TAVI in inoperable patients of the PARTNER-B trial, which make it unethical to deny TAVI in these patients. This will lead to serious selection bias in registry data. In that case, using a historical cohort of ST patients, for example as in Freeman et al. [35], might better reflect real-world outcomes in ST.

### Limitations

This study has several limitations. Firstly, we experienced difficulties in using the Philips checklist to assess the methodological quality of the studies. Some criteria are umbrella-criteria that should be assessed differently for different types of data (i.e. utilities, costs, etc). For many criteria the methods were described but not explained or justified. In these cases we decided that the study fulfilled the criteria but we added a remark that there was no justification reported. Sometimes criteria were partially fulfilled which made it difficult to decide if the criteria should be assessed as fulfilled or not. Therefore, we did not exclude studies with low scores on the Philips checklist. Secondly, it was often difficult to fully understand the details of a decision-analytic model because of space limits on papers.

## Conclusion

This review provided an overview of the existing decision-analytic models regarding the cost-effectiveness of heart valve implantations. Our results showed that in most studies TAVI was cost-effective compared to ST in inoperable and high-risk operable patients and in all studies SAVR was cost-effective compared to ST in operable patients, but the results were not consistent on the cost-effectiveness of TAVI versus SAVR in high-risk operable patients. This review showed that future models can improve their methodological quality and that there is room for patient-simulation models considering the cost-effectiveness of heart valve implantations in other valve positions besides the aortic valve, performed from a societal perspective.

## Electronic supplementary material

Below is the link to the electronic supplementary material.

Supplementary material 1 (PDF 305 kb)
