# Supplementary material for: Systematic review of model-based economic evaluations of heart valve implantations
Source: Eur J Health Econ. 2017 Mar 6;19(2):241–55. doi: 10.1007/s10198-017-0880-z (PMC5813051; doi:10.1007/s10198-017-0880-z)
Supplement: Supplementary file 1 — Supplementary material 1 (PDF 305 kb) [file 10198_2017_880_MOESM1_ESM.pdf]

Systematic review of model-based economic evaluations of heart valve implantations: Supplementary material

Simone A. Huygens, MSc[1,2], Johanna. J.M. Takkenberg, MD, PhD[1], Maureen P.M.H. Rutten-van Mölken, PhD[2]

1. Department of Cardiothoracic Surgery, Erasmus University Medical Center, Rotterdam, the Netherlands. PO Box 2040. 3000 CA Rotterdam, The Netherlands.
2. Institute for Medical Technology Assessment / Department of Health Policy and Management, Erasmus University, Rotterdam, the Netherlands. Bayle Building - Campus Woudestein. PO Box 1738. 3000 DR Rotterdam, The Netherlands.

Corresponding author: Simone A. Huygens, MSc. E-mail: s.huygens@erasmusmc.nl

European Journal of Health Economics

Appendix 1 – Search strategy literature review per database

Embase.com
('economic aspect'/exp OR (cost* OR econom* OR price):ab,ti) AND ('decision tree'/de OR 'decision support system'/de OR model/exp OR algorithm/de OR (model* OR (decisi* NEAR/3 (tree* OR rule* OR support* OR system*)) OR (concept* NEAR/3 framework*) OR simulat* OR algorithm*):ab,ti) AND ('heart valve'/de OR 'heart valve surgery'/de OR 'heart valve prosthesis'/exp OR 'heart valve replacement'/exp OR 'aorta valve'/de OR 'pulmonary valve'/de OR 'aorta stenosis'/exp OR 'heart valve stenosis'/de OR 'heart valve regurgitation'/de OR 'valvular heart disease'/de OR 'aorta valve disease'/exp OR 'pulmonary valve disease'/exp OR (((heart OR cardiac OR aort* OR pulmonar*) NEAR/3 valv*) OR ((aort* OR pulmonar*) NEAR/3 (steno* OR calcif* OR regurgitat*)) OR aortostenos*):ab,ti) NOT ([animals]/lim NOT [humans]/lim)

Medline (OvidSP)
(((exp "economics"/ OR economics.xs. OR (cost* OR econom* OR price).ab,ti.) AND ("decision trees"/ OR "Decision Support Systems, Clinical"/ OR exp "Models, Theoretical"/ OR algorithms/ OR (model* OR (decisi* ADJ3 (tree* OR rule* OR support* OR system*)) OR (concept* ADJ3 framework*) OR simulat* OR algorithm*).ab,ti.)) OR exp "Models, Economic"/) AND ("heart valves"/ OR "Heart Valve Prosthesis"/ OR "Heart Valve Prosthesis Implantation"/ OR "Aortic Valve"/ OR "pulmonary valve"/ OR exp "Aortic Valve Stenosis"/ OR "Heart Valve Diseases"/ OR (((heart OR cardiac OR aort* OR pulmonar*) ADJ3 valv*) OR ((aort* OR pulmonar*) ADJ3 (steno* OR calcif* OR regurgitat*)) OR aortostenos*).ab,ti.) NOT (exp animals/ NOT humans/)

Cochrane
((cost* OR econom* OR price):ab,ti) AND ((model* OR (decisi* NEAR/3 (tree* OR rule* OR support* OR system*)) OR (concept* NEAR/3 framework*) OR simulat* OR algorithm*):ab,ti) AND ((((heart OR cardiac OR aort* OR pulmonar*) NEAR/3 valv*) OR ((aort* OR pulmonar*) NEAR/3 (steno* OR calcif* OR regurgitat*)) OR aortostenos*):ab,ti)

Web-of-science
TS=(((cost* OR econom* OR price)) AND ((model* OR (decisi* NEAR/3 (tree* OR rule* OR support* OR system*)) OR (concept* NEAR/3 framework*) OR simulat* OR algorithm*)) AND ((((heart OR

cardiac OR aort* OR pulmonar*) NEAR/3 valv*) OR ((aort* OR pulmonar*) NEAR/3 (steno* OR calcif* OR regurgitat*)) OR aortostenos*)))

Scopus
TITLE-ABS-KEY(((cost* OR econom* OR price)) AND ((model* OR (decisi* W/3 (tree* OR rule* OR support* OR system*)) OR (concept* W/3 framework*) OR simulat* OR algorithm*)) AND ((((heart OR cardiac OR aort* OR pulmonar*) W/3 valv*) OR ((aort* OR pulmonar*) W/3 (steno* OR calcif* OR regurgitat*)) OR aortostenos*)))

PubMed publisher
((((cost*[tiab] OR econom*[tiab] OR price[tiab])) AND ((model*[tiab] OR (decisi*[tiab] AND (tree*[tiab] OR rule*[tiab] OR support*[tiab] OR system*[tiab])) OR (concept*[tiab] AND framework*[tiab]) OR simulat*[tiab] OR algorithm*[tiab])))) AND ((((heart[tiab] OR cardiac[tiab] OR aort*[tiab] OR pulmonar*[tiab]) AND valv*[tiab]) OR ((aort*[tiab] OR pulmonar*[tiab]) AND (steno*[tiab] OR calcif*[tiab] OR regurgitat*[tiab])) OR aortostenos*[tiab])) AND publisher[sb]

Google Scholar
Economy|Economic|economical|economically|economics|cost|costs model|algorithm|"decision tree|rule|support|system"|"conceptual framework"|simulation "aorta|aortic|heart|cardiac|pulmonary valve|valves|stenosis|regurgitation"

Appendix 2: Supplementary tables

	SHTG 2010	Gada 2012a	Gada 2012b	Neyt 2012	Watt 2012	Beresniak 2013	Doble 2013	Fairbairn 2013	Hancock-Howard 2013	Murphy 2013	Orlando 2013	Queiroga 2013	Simons 2013	Brecker 2014	**Total**	%
<u>Structure</u>																
Statement of decision problem and objective																
1. Is there a clear statement of the **decision problem**?	1	1	1	1	1	1	1	1	1	1	1	1	1	1	**14**	100
2. Is the **objective** of the evaluation and model specified and consistent with the stated decision problem?	1	1	1	1	1	1	1	1	1	1	1	1	1	1	**14**	100
3. Is the primary **decision-maker** specified?	1	0	0	1	1	1	1	1	1	1	1	1	0	1	**11**	79
Statement of scope and perspective																
4. Is the **perspective** of the model stated clearly?	1	1	1	1	1	1	1	1	1	1	1	1	1	1	**14**	100
5. Are the **model inputs** consistent with the stated perspective?	1	1	1	1	1	1	1	1	1	1	1	1	0	1	**13**	93
6. Has the **scope** of the model been stated and justified?	1	1	1	1	1	1	1	1	1	1	1	1	1	1	**14**	100
7. Are the **outcomes** of the model consistent with the perspective, scope and overall objective of the model?	1	1	1	1	1	1	1	1	1	1	1	1	1	1	**14**	100
Rationale for structure																
8. Is the structure of the model consistent with a **coherent theory of the health condition** under evaluation?	1	1	1	1	1	1	1	0	1	1	1	0	1	1	**12**	86
9. Are the **sources of data** used to develop the structure of the model specified?	0	0	0	0	1	0	0	0	0	0	0	0	1	1	**3**	21
10. Are the **causal relationships** described by the model structure justified appropriately?	1	1	1	1	1	1	1	1	1	1	1	1	1	1	**14**	100
Structural assumptions																
11. Are the structural assumptions **transparent and justified**?	1	1	1	1	1	1	1	1	1	1	1	1	1	1	**14**	100
12. Are the structural assumptions **reasonable** given the overall objective, perspective and scope of the model?	0	0	0	1	0	1	1	0	1	1	0	1	1	1	**8**	57
Strategies and comparators																
13. Is there a clear **definition of the options** under consideration?	1	1	1	1	1	1	1	1	1	1	1	1	1	1	**14**	100
14. Have **all feasible and practical options** been evaluated?	1	1	1	1	1	1	1	1	1	1	1	1	1	1	**14**	100
15. Is there **justification for the exclusion** of feasible options?	-	-	-	-	-	-	-	-	-	-	-	-	-	-	**-**	-
Model type																
16. Is the chosen **model type** appropriate given the decision problem and specified causal relationships within the model?	1	1	1	1	1	1	1	1	1	1	1	1	1	1	**14**	100

	SHTG 2010	Gada 2012a	Gada 2012b	Neyt 2012	Watt 2012	Beresniak 2013	Doble 2013	Fairbairn 2013	Hancock-Howard 2013	Murphy 2013	Orlando 2013	Queiroga 2013	Simons 2013	Brecker 2014	**Total**	%
Time horizon																
17. Is the **time horizon** of the model sufficient to reflect all important differences between options?	1	1	1	1	1	1	1	1	0	1	1	0	1	0	**11**	79
18. Are the time horizon of the model, the duration of treatment and the duration of treatment effect **described and justified**?	1	0	0	1	1	1	1	1	1	1	1	0	1	1	**11**	79
Disease pathways																
19. Do the disease states (state transition model) or the pathways (decision tree model) **reflect the underlying biological process** of the disease in question and the impact of the intervention?	1	1	1	1	1	1	1	1	1	1	1	0	1	1	**13**	93
Cycle length																
20. Is the **cycle length** defined and justified in terms of the natural history of the disease?	1	1	1	1	1	0	1	1	-	1	-	1	1	1	**11**	92
Data																
Data identification																
21. Are the **data identification methods** transparent and appropriate given the objectives of the model?	1	0	0	1	1	0	1	1	1	1	1	1	1	1	**11**	79
22. Where **choices** have been made **between data sources**, are these justified appropriately?	0	0	0	0	1	0	0	1	1	1	1	0	0	1	**6**	43
23. Has **particular attention** been paid to identifying data for the **important parameters** in the model?	1	0	0	1	1	0	1	1	1	1	1	1	1	1	**11**	79
24. Has the **quality of the data** been assessed appropriately?	1	0	1	1	1	0	1	1	1	1	1	1	1	1	**12**	86
25. Where **expert opinion** has been used, are the methods described and justified?	0	-	-	0	0	-	-	-	0	-	0	0	-	-	**0**	0
Data modelling																
26. Is the data modelling methodology based on **justifiable statistical and epidemiological techniques**? (specific issues to consider include those listed under D2a-d, below)	1	1	1	1	1	1	1	1	1	1	1	1	1	1	**14**	100
Baseline data																
27. Is the **choice of baseline data** described and justified?	1	1	1	1	1	1	1	1	1	1	1	1	1	1	**14**	100
28. Are **transition probabilities** calculated appropriately?	1	1	1	1	0	0	1	0	0	1	1	0	1	1	**9**	64
29. Has a **half-cycle correction** been applied to both cost and outcome?	-	-	-	0	-	-	0	-	-	-	-	0	-	0	**0**	0
30. If not, has this omission been justified?	-	-	-	0	-	-	0	-	-	-	-	0	-	0	**0**	0

	SHTG 2010	Gada 2012a	Gada 2012b	Neyt 2012	Watt 2012	Beresniak 2013	Doble 2013	Fairbairn 2013	Hancock-Howard 2013	Murphy 2013	Orlando 2013	Queiroga 2013	Simons 2013	Brecker 2014	**Total**	%
Treatment effects																
31. If relative treatment effects have been derived from **trial data**, have they been **synthesised** using appropriate techniques?	-	0	0	-	-	-	-	-	-	-	-	-	-	-	**0**	0
32. Have the methods and assumptions used to **extrapolate short-term results to final outcomes** been documented and justified?	1	0	0	1	1	0	1	1	1	1	1	1	1	1	**11**	79
33. Have alternative assumptions been explored through sensitivity analysis?	0	0	0	0	1	0	1	0	0	0	0	0	1	1	**4**	29
34. Have assumptions regarding the **continuing effect of treatment once treatment is complete** been documented and justified?	1	0	0	1	0	0	1	1	1	1	1	0	1	1	**9**	64
35. Have alternative assumptions been explored through sensitivity analysis?	0	1	1	1	1	0	1	1	0	1	1	0	0	1	**9**	64
Costs																
36. Are the **costs** incorporated in the model justified?	1	1	1	1	1	1	1	1	1	1	1	1	1	1	**14**	100
37. Has the **source** for all costs been described?	1	1	1	1	0	0	1	1	1	0	1	0	1	1	**10**	71
38. Have **discount rates** been described and justified given the target decision-maker?	1	0	0	1	1	0	1	1	1	0	1	1	1	1	**10**	71
Quality of life weights (utilities)																
39. Are the **utilities** incorporated into the model appropriate?	1	1	1	1	1	-	1	1	1	1	1	-	1	1	**12**	100
40. Is the **source** for the utility weights referenced?	1	1	1	1	1	-	1	1	1	1	1	-	1	1	**12**	100
41. Are the methods of **derivation for the utility weights** justified?	0	1	1	1	0	-	0	1	1	0	0	-	1	0	**6**	50
Data incorporation																
42. Have all **data** incorporated into the model been **described and referenced** in sufficient detail?	0	0	0	1	0	0	1	1	1	0	1	0	0	1	**6**	43
43. Has the use of **mutually inconsistent data** been justified (i.e. are assumptions and choices appropriate)?	-	-	-	1	-	-	-	-	-	-	-	-	-	1	**2**	100
44. Is the **process of data incorporation** transparent? (i.e. Is it clear whether data are incorporated as point estimate or distribution (+ justification for choice of distribution)?)	1	0	1	1	1	0	1	1	0	1	1	0	1	1	**10**	71
45. If data have been incorporated as **distributions,** has the choice of distribution for each parameter been described and justified?	0	1	1	1	0	0	1	0	0	1	1	1	1	1	**9**	64
46. If data have been incorporated as distributions, is it clear that **second order uncertainty is reflected**?	1	1	1	1	1	-	1	1	-	1	1	1	1	1	**12**	100

	SHTG 2010	Gada 2012a	Gada 2012b	Neyt 2012	Watt 2012	Beresniak 2013	Doble 2013	Fairbairn 2013	Hancock-Howard 2013	Murphy 2013	Orlando 2013	Queiroga 2013	Simons 2013	Brecker 2014	**Total**	%
Assessment of uncertainty																
47. Have the **four principal types of uncertainty** been addressed?	0	0	0	1	0	0	0	0	0	0	0	0	0	0	**1**	7
48. If not, has this omission of particular forms of uncertainty been justified?	0	0	0	-	0	0	0	0	0	0	0	0	0	0	**0**	0
49. Have **methodological uncertainties** been addressed by running alternative versions of the model with different methodological assumptions?	0	0	0	1	0	0	0	1	0	0	0	1	0	0	**3**	21
50. Is there evidence that **structural uncertainties** have been addressed via sensitivity analysis?	0	0	0	1	1	1	1	1	1	0	0	1	0	1	**8**	57
51. Has **heterogeneity** been dealt with by running the model separately for different subgroups?	0	0	0	1	0	0	0	0	0	0	1	0	1	1	**4**	29
52. Are the methods of assessment of **parameter uncertainty** appropriate?	1	1	1	1	0	1	1	1	1	1	1	1	1	1	**13**	93
53. If data are incorporated as **point estimates**, are the **ranges used for sensitivity analysis** stated clearly and justified?	-	0	0	1	1	0	1	1	1	0	1	1	1	1	**9**	69
<u>Consistency</u>																
Internal consistency																
54. Is there evidence that the **mathematical logic of the model** has been tested thoroughly before use?	1	0	0	0	0	0	1	0	0	0	0	0	1	0	**3**	21
External consistency																
55. Are any **counterintuitive results** from the model explained and justified?	-	-	-	-	-	-	-	-	-	-	-	-	-	-	**-**	-
56. If the model has been **calibrated against independent data**, have any differences been explained and justified?	0	0	0	1	0	0	0	0	0	0	0	0	1	0	**2**	14
57. Have the results of the model been **compared with those of previous models** and any differences in results explained?	-	-	-	1	0	-	1	1	1	0	1	0	1	1	**7**	70
Total score (not corrected for N/A)	34	27	29	46	35	22	42	39	35	35	39	28	41	44		
Number of items not applicable	8	7	7	4	6	12	5	7	8	7	7	7	7	4		
**Total score, % (corrected for N/A)**	**69**	**54**	**58**	**87**	**69**	**49**	**81**	**78**	**71**	**70**	**78**	**56**	**82**	**83**		

Table A2.1. Philips checklist. 1 = criteria is fulfilled, 0 = criteria is not fulfilled, and '-' criteria is not applicable (N/A).

Explanation for underlined scores:

17. Brecker: Time horizon in base-case analysis is 5 years, which is not equivalent to lifetime in this patient group. However, a sensitivity analysis with a time horizon of 10 years (=equivalent to lifetime) was performed. Neyt: This criteria is fulfilled in the subgroup of inoperable patients, but not for the subgroup of high-risk operable patients where the time horizon was only one year.

19. Watt and Brecker: Health states based on location of care, reoperations and post-hospital rehabilitations, therefore it is only clear that they consider reoperations as complications. Faibairn and Simons: NYHA classes are not appropriate health states.

22. Hancock-Howard: Only for the data sources on costs.

29-30: Brecker, Neyt and Queiroga: Not reported, therefore unclear if this was done.

41. Neyt: This criteria is fulfilled in the subgroup of inoperable patients (where utilities are based on EQ-5D measurements), but not for the high-risk operable group.

45. Queiroga: Distribution only defined for costs.

Author	Mortality	Baseline clinical data	Resource use	Costs	Utilities
**SHTG 2010**	Revive trials	Revive trials	NR	Previously published studies	NYHA class utilities (39) applied to Revive trials NYHA class proportions
**Gada 2012a**	Existing registries	Existing registries	Previously published studies	Previously published studies	PARTNER trial, measured with EQ-5D
**Gada 2012b**	Existing registries	Existing registries	Previously published studies	Previously published studies	PARTNER trial, measured with EQ-5D
**Neyt 2012**	PARTNER trial	PARTNER trial	Aggregated cost data of TAVI patients and data on hospital stays after surgical AVR complemented with APR-DRG costs	Aggregated cost data of TAVI patients and data on hospital stays after surgical AVR complemented with APR-DRG costs	Measured with EQ-5D in PARTNER trial for inoperable patients and based on assumptions for high-risk operable patients
**Watt 2012**	PARTNER trial	PARTNER trial	Expert opinion and literature review	British National Formulary, other publicly available national databases	NYHA class utilities (40) applied to PARTNER trial NYHA class proportions
**Beresniak 2013**	Cohort study	NR	Resource utilization survey	French medical information system program using national charge table and DRG coding	N/A
**Doble 2013**	PARTNER trial	PARTNER trial	NR	Ontario Case Costing Initiative	NYHA class utilities (37) applied to PARTNER trial NYHA class proportions
**Fairbairn 2013**	PARTNER trial	PARTNER trial	NR	NHS tariff payment by results fee and further costs calculated based on previously published hospitalization annual hazard per NYHA category	NYHA class utilities (38) applied to PARTNER trial NYHA class proportions combined with additional utility decrements for complications
**Hancock-Howard 2013**	PARTNER trial	PARTNER trial	Ontario Health Insurance Plan fee schedule and the Ontario Case Costing Initiative database	Ontario Health Insurance Plan fee schedule, the Ontario Case Costing Initiative database, and adjusted from French economic model	PARTNER trial, measured with EQ-5D
**Murphy 2013**	PARTNER trial	PARTNER trial	Previously published studies	Previously published studies	NYHA class utilities (39) applied to PARTNER trial NYHA class proportions
**Orlando 2013**	PARTNER trial	NR	NR	South Central report, NHS reference costs and previously published studies	NYHA class utilities (39) applied to PARTNER trial NYHA class proportions

Author	Mortality	Baseline clinical data	Resource use	Costs	Utilities
**Queiroga 2013**	PARTNER trial	PARTNER trial	Based on data from a pre-planned economic study, conducted in parallel with the PARTNER trial	Based on data from a pre-planned economic study, conducted in parallel with the PARTNER trial	N/A
**Simons 2013**	PARTNER trial	PARTNER trial	NR	Medicare data and previously published studies	Medical Expenditure Panel Survey data
**Brecker 2014**	ADVANCE and PARTNER trial	ADVANCE and PARTNER trial	ADVANCE trial	British National Formulary, other publicly accessible databases and previously published studies	ADVANCE trial, measured with EQ-5D(48) (TAVI) and NYHA class utilities (40) applied to PARTNER trial NYHA class proportions (ST)

Table A2.2. Data sources and outcomes. NR: Not reported. **APR-DRG:** all patient refined diagnosis related groups. **ICU:** intensive care unit. **SAVR:** surgical aortic valve replacement. **TAVI:** transcatheter aortic valve replacement. **TF:** transfemoral. **TA:** transapical. **MM:** medical management. **ST:** standard therapy; including MM and/or balloon aortic valvuloplasty (BAV). **NYHA class:** New York Heart Association class. **PARTNER:** Placement of Aortic Transcatheter Valves.(3,4) **REVIVE:** The Registry of Endovascular Implantation of Valves in Europe trial started in 2003 in a single centre in France with the aim to study the feasibility and safety of TAVI in inoperable patients.(47) **ADVANCE:** Multicentre, non-randomized study that included 44 centres in 12 countries evaluating the outcomes of a self-expanding transcatheter aortic valve system in patients considered inoperable or at a higher surgical risk.(48)
